# Different Effects of Adding White Noise on Cognitive Performance of Sub-, Normal and Super-Attentive School Children

**DOI:** 10.1371/journal.pone.0112768

**Published:** 2014-11-13

**Authors:** Suzannah K. Helps, Susan Bamford, Edmund J. S. Sonuga-Barke, Göran B. W. Söderlund

**Affiliations:** 1 Institute for Disorders of Impulse and Attention, Department of Psychology, University of Southampton, Southampton, United Kingdom; 2 Faculty of Teacher Education and Sports, Sogndal University College, Sogndal, Norway; 3 Department of Experimental Clinical & Health Psychology, University of Ghent, Ghent, Belgium; University of L’Aquila, Italy

## Abstract

**Objectives:**

Noise often has detrimental effects on performance. However, because of the phenomenon of stochastic resonance (SR), auditory white noise (WN) can alter the “signal to noise” ratio and improve performance. The Moderate Brain Arousal (MBA) model postulates different levels of internal “neural noise” in individuals with different attentional capacities. This in turn determines the particular WN level most beneficial in each individual case–with one level of WN facilitating poor attenders but hindering super-attentive children. The objective of the present study is to find out if added WN affects cognitive performance differently in children that differ in attention ability.

**Methods:**

Participants were teacher-rated super- (N = 25); normal- (N = 29) and sub-attentive (N = 36) children (aged 8 to 10 years). Two non-executive function (EF) tasks (a verbal episodic recall task and a delayed verbal recognition task) and two EF tasks (a visuo-spatial working memory test and a Go-NoGo task) were performed under three WN levels. The non-WN condition was only used to control for potential differences in background noise in the group testing situations.

**Results:**

There were different effects of WN on performance in the three groups-adding moderate WN worsened the performance of super-attentive children for both task types and improved EF performance in sub-attentive children. The normal-attentive children’s performance was unaffected by WN exposure. The shift from moderate to high levels of WN had little further effect on performance in any group.

**Significance:**

The predicted differential effect of WN on performance was confirmed. However, the failure to find evidence for an inverted U function challenges current theories. Alternative explanations are discussed. We propose that WN therapy should be further investigated as a possible non-pharmacological treatment for inattention.

## Introduction

Under most circumstances, information processing is disturbed by environmental noise and other non-task compatible distractors [1], [2]. Children with attention problems are, under many conditions, especially vulnerable to distraction e.g. [3]. However, researchers have recently reported that under certain circumstances individuals with attention problems appear to benefit from the addition of specific forms of environmental noise. Typically, this facilitative effect has been limited to non-vocal background music on simple arithmetic task performance, [4], [5] but Stansfeld et al. [6] found just that under certain conditions even road traffic noise can improve performance on episodic memory tasks, particularly in children at risk of attention problems and academic under-achievement.

Furthermore, Söderlund et al. [7] have demonstrated that adding auditory white noise (WN) to the environment enhanced the memory performance of children with ADHD-type problems but disrupted that of non-ADHD control children. These effects were replicated in a second study for children with sub-clinical attention problems [8]. These results raise two obvious questions. First, how or by what mechanism does WN improve performance? Second, why does the same level of WN have such apparently different effects on children with different levels of attentional problems?

The exact mechanism behind WN benefits is not yet known. It has been proposed that random noise enhances neural communication via the phenomenon of stochastic resonance (SR). The phenomenon of SR is observed when an increase in the level of unpredictable fluctuations, e.g. WN, causes an increase in a metric of the quality of signal transmission or signal detection, or in other words increases the signal-to-noise ratio. SR is usually quantified by plotting signal detection, or in this study cognitive performance, as a function of WN intensity. The SR effect appears highly sensitive to both the intensity of the signal and the noise level; this relationship follows an inverted U-curve function, where performance peaks at moderate noise levels. This means that a moderate level of WN is beneficial for performance whereas too little does not add the power required to bring the signal over the threshold and too much overpowers the signal, leading to a deterioration in attention and performance [9], [10]. Signaling in the brain is characterized by noisy inputs and outputs. The task of the central nervous system is to distinguish between the signal, the information-carrying component, and noise (i.e. meaningless neural inputs that interfere with the signal). However, noise is an integral part of interneuronal communication and a sufficient amount of noise may be necessary for the normal functioning of the nervous system [11], [12]. This in turn modulates neural synchronization whereby particular brain regions sub-serving specific functions establish transient networks that accomplish perception, cognition, or action [13]. Thus, it has been shown that random noise enhances detection of weak sensory signals like hearing [14], touch [15], vision [16] through SR. “Touch WN” improved vibrotactile sensitivity in healthy young people [15], vibrating soles improved motor performance in elderly [17], in stroke patients [18], and those with Parkinson’s disease [19]. High-level performance can also be improved by WN (e.g., face recognition [20] and arithmetic computations (77 dB) [21]). Moreover, SR can work across modalities such that detection of weak visual signals improved considerably when exposed to high levels of auditory noise (75 dB) [22]. To sum up, the concept of SR attempts to explain the paradox that the brain seems to utilize WN to differentiate the signal in the targeted stimuli from non-target noise [23]. WN accordingly improves or increases the signal-to-noise ratio.

How can we explain individual differences in WN effects? Sikström & Söderlund [24] proposed that individual differences arise because each person has a certain level of background “noise” intrinsic to his or her neural system associated with neurotransmitter function. Dopamine was hypothesized to be especially important because it modulates the neural cell’s response to the environment and determines the probability that it will fire an action potential following the presentation of salient stimuli [25]. Dopamine function is related to individual differences in attention [26] and cognition [27]. Dysfunction is found among ADHD patients [28]. Stimulant medication, acting via the dopamine system, reduces symptoms of inattention and improves cognitive performance within normal populations [29], [30] and in patients with an ADHD diagnosis [31], [32]. In the Moderate Brain Arousal Model (MBA); [24] internal neural noise and external WN are hypothesized to act additively in relation to SR. Thus, where there are low levels of neural noise, i.e. low continuous dopamine activity, more external WN is required for SR to occur. The facilitative SR effect is predicted to demand high levels of WN where internal noise levels are low, but where high internal noise levels are present, i.e. high continuous dopamine activity, less external WN will be required. Low accuracy in neural communication is associated with low levels of extracellular dopamine distinguished by neurons firing at random causing inattention and in accordance with Servan-Schreiber et al.’s [25] terminology this is a “low gain” state. From this one can conclude that WN benefit only occurs when a nervous system is not working at its optimum [23], [33]. This leads to the prediction that inattentive children will benefit more from higher levels of environmental WN than attentive children, for whom such noise levels will have a detrimental effect on performance. Moreover, the literature describes two kinds of WN facilitation: threshold SR and supra threshold SR (SSR) – differentiated by the nature of the relationship between the strength of the signal and the noise required for SR to occur [23], [34]. For example, in auditory threshold SR the signal should be presented just below the hearing threshold (20–35 dB, depending on age and frequency) and the noise should be within the same range (20–35 dB) for SR to occur. In supra threshold SR this will occur when all noises added equal the signal mean amplitude [34], [35]. This means that both signal and noise can be far above the hearing threshold. The present study focuses on supra-threshold SR using a lowest WN level of 65 dB.

The goal of the current study was to test the hypothesis that different intensities of WN will exert differential effects on children with different levels of attention-ability through the differential action of SR in a way predicted by the MBA hypothesis. Despite the current categorical approach to the diagnosis of ADHD, attentional problems appear to display a dimensional rather than a categorical structure [36]. This conclusion is supported by taxometric studies that have failed to find evidence for an attention deficit taxon [37], [38] as well as behavioral genetic studies that suggest heritability is similar across different ranges of symptom severity [39]. Different severities of inattentiveness, distractibility, and associated academic problems are distributed quasi-normally throughout populations. Children who do not meet the full ADHD criteria may still suffer significant impairment. In fact, there are a number of implications of this conceptualization of attention problems for the current study. In particular it means that the comparison of the moderating effect of attentional abilities on the effects of WN on performance should not be limited to a binary comparison of attentive vs. inattentive or ADHD vs non-ADHD children. From a dimensional perspective the comparison of WN effects across sub-attentive vs. normal attentive vs. super-attentive children would be most appropriate. Assuming that the mechanisms governing the link between attentiveness, internal noise and external WN noise hold across the full range of attentional abilities we would predict different levels of WN being optimal for these different groups of children. Consistent with the MBA hypothesis we predict that the inverted U function of WN is right shifted for sub-attentive children and left-shifted for super-attentive children compared to children with normal attention. The past focus on binary conceptualizations of attention problems has meant that no studies, as far as we are aware, have examined either the neuro-psychological profiles of super-attentive children or the impact of environmental context on performance.

Our specific predictions are as follows: (i) in general, super-attentive children will have superior task performance compared to normal attentive children who will in turn out-perform sub-attentive children; (ii) moderate to high levels of WN will have facilitative effects on attention and related task performance for sub-attentive children but will disrupt performance in super-attentive children with normal attentive children lying somewhere in between. To test these predictions we extended previous studies of WN, attention and performance by: i) comparing children with normal-, sub-, and super- attention ability as defined by teachers; ii) extending the set of signal-to-noise ratios tested by studying multiple WN levels and observing the effect on performance and iii) exploring the generalization of the WN effect across a range of different tasks. Specifically, the original WN studies employed a verbal memory task [7], [8]. However, tests of executive functioning (EF) have been more heavily implicated in studies of attention deficits and have been shown to differentiate inattentive from attentive children [40]. Therefore, in addition to the memory task employed in the original study, we include two EF measures in our test battery.

## Method

### Study recruitment and screening

Ethical approval was obtained from the University of Southampton Psychology Ethics Committee. Written consent was obtained from the school’s head teacher on behalf of the children, and written assent was obtained from the children themselves. Prior to the study start, parents were sent information forms about the study and were given the option to opt their children out of the study. Copies of the written consent/assent forms are stored in a locked filing cabinet. This consent procedure was approved by the University of Southampton Psychology Ethics Committee. All children were recruited from a single local junior school; children from school years 4 and 5 (aged 8 to 10 years) and their parents were sent information letters and, in accordance with the head teachers’ preferences, were given the option to opt-out of the study. Only one child did so. All remaining year 4 and 5 children (N = 150∶58 boys, 92 girls) were screened for levels of attention at school using the teacher-report SWAN rating scale [41]. The SWAN has 18 items probing attention and behavior. On the basis of the teacher-ratings on the attention items of this scale, 36 sub-attentive (bottom 20 percent of scores), 29 average (middle 20 percent), and 25 super-attentive (top 20 percent) children were selected for the study. The groups were matched for age, but were unable to be matched for gender as very few boys were rated in the super-attentive range (only 2 boys out of the 150 screened children were rated as being in the top quintile for attention).

### Test Battery

Four laboratory tests, two EF and two non-EF tasks, including the memory task used in the original study [7], were employed. These tasks were presented in a fixed order; participants completed them in small groups (3–4) on individual laptops in a quiet classroom with two experimenters present.

### Non-EF verbal memory tasks

#### i) Verbal episodic memory task (Word recall; 5 minutes; [8])

Lists of nouns were presented to the participants in the auditory mode using a laptop. Participants were asked to remember as many nouns as possible. Two lists of 10 nouns (ISI 5 seconds) were presented in each WN condition: five of these words were low frequency words (frequency <100 per million) and five high frequency words (frequencies >200 per million: as determined by the children’s printed word database [42]). Each list was matched for word frequency, word length and syllable number. Immediately after each list, participants were asked to perform a written free recall test. Balanced Latin squares were used to ensure that each word list was equally likely to be heard in each noise condition, and within each list, words were presented in a random order to each child.

#### ii) Verbal recognition task (Word recognize; 5 minutes)

This task tested the recognition of the words presented in the verbal episodic memory task above. The 20 words presented in the verbal episodic memory task and 20 other words (matched to the initial lists on frequency, word length and number of syllables) were presented in the auditory modality via a laptop. Participants were required to indicate whether a word had been presented in the previous task by pressing symbols of either a tick or a cross on computer keyboard. The ISI was 3 seconds and words were presented in a random order for each child.

### EF tasks

#### i) Visuo-spatial working memory test (Spanboard; [43]; 5 minutes)

Participants were asked to remember the location of dots that appeared in a 4×4 grid (16 squares) presented on a computer screen and to recall this sequence using the computer mouse to click the correct grid locations. In the first trial, the array consisted of two dots (ISI was 3 seconds, 2250 ms dot exposure, 750 ms pause). On every second successive trial, one dot was added until the participant made an error in both trials on that particular level.

#### ii) The Go/No-Go Task [44]

The Go/No-Go task required a motor response (pressing the right or left mouse button) to either be selectively executed or inhibited depending on whether a Go (left/right green arrow: 75% trials) or No-Go (double-ended green arrow: 25% trials) stimulus appeared on the computer screen. The inter-stimulus interval (ISI) was 1500 ms: A 100 ms stimulus duration followed by a blank screen for 1400 ms. For the purposes of the current analysis the dependent variable for each of these tasks was the number of correct responses.

#### Simple two-choice RT task (2-CR RT) [45]

This task was used to validate the attentional ability groupings and so was presented under normal background noise (NBN) condition only. Participants responded to a computer presentation of a green target arrow that pointed left or right. The target arrow was presented in the center of the computer monitor. Participants responded, by pressing a right or left keyboard button, to indicate the direction of each arrow. Each trial lasted 1500 ms (stimulus presentation time 400 ms, inter-stimulus interval 1100 ms). The task duration was 5 minutes and a total of 200 trials were presented. Dependent variables were number of omission and commission errors.

### Experimental Design

Each task was performed under three WN levels. Using high quality headphones participants received WN separately in each ear. The WN was mixed in adobe audition and was in phase across ears. Output signal was measured with a standard dB meter. For the two EF tasks these levels were 65 dB, 75 dB and 85 dB. The non-EF tasks were performed under slightly lower WN levels −65 dB, 70 dB and 75 dB. This was necessary as the words were not audible when louder WN levels were used. The order in which noise levels were presented was counterbalanced using a Latin Square. For all tasks the performance was also measured under normal background noise conditions to allow the effect of different levels of background noise on performance, which may vary as a function of time of day and classroom setting, to be controlled in the analyses.

### Analytical strategy

All outliers (>2 SD from the group mean score for each condition) and any missing data were replaced with the group mean for that condition. Data was replaced to the following extent: Go/No-Go 3.1% of; Spanboard 2.5%; Word recall 4.9%; and Word recognition 5.8%. Noise in the data set was not caused by individuals and outliers were spread across participants. The SWAN groupings were validated by comparing performance on the four experimental tasks and the simple two choice reaction time tasks in the NBN condition using a one-way ANOVA. We examined the correlations between EF and non-EF measures with a view to combining them to create two dependent variables. Because a different range of WN levels were used for EF and non-EF tasks we first ran two separate ANOVAs looking at the effects of noise level (65 dB vs 75 dB vs 85 dB for EF tasks; 65 dB vs 70 dB vs 75 dB for non-EF tasks) and group (sub-, vs normal- vs super-attentive) on EF and non-EF performance. We also ran a single three way ANOVA using the two noise levels that were common for tasks: group (sub-vs normal vs super-attentive), noise level (65 dB vs75 dB) and task type (EF vs non-EF) were included as factors. Thereafter we ran separate two-way ANOVAS for all four tasks to control for deviations within the EF and non-EF tasks. In all analyses performance under normal background noise was used as a covariate. This was done because the normal ambient noise at schools was not constant and varied considerably during the day, between test occasions, and between schools, dB values could range from 45–65 dB. The no-noise condition was used as covariate for all separate ANOVA analyses as well. Gender was also added as covariate to control for possible effects of gender on the dependent variable. This did not change data significantly, which is why these figures are not reported in the result section.

## Results

Table 1 reports the performance on the five tasks in the normal background noise condition for the three groups. On all tasks there was a large and significant effect of group. More specifically, the sub-attentive group always performed less well than the average attentive group, with the super-attentive group performing the best. The differences between sub- and average/super attentive children were significant for all tasks. The super-attentive children were significantly better than the average attentive children only on the two EF tasks. These effects were unchanged when age, gender and school performance were added as covariates. The correlational analysis supported the combining of the EF (*r* = .222, *p*<.001) and non-EF task pairs (*r* = .276, *p*<.001) to give two measures. These were created using the factor loadings obtained from factor analysis as weights. Factor loadings for each were as follows: GNG.661; Spanboard.558; Word recall.742; Word recognition.723. However, while the correlation only had a moderate strength, the effects of noise levels in all tasks will be presented separately as well.

**Table 1:** Group characteristics – Sub-attentive (Sub), Normal-attention (Normal) and Super-attentive groups (Super).

	*Sub (N = 36) Mean (SD)*	*Normal(N = 29) Mean (SD)*	*Super(N = 25) Mean(SD)*	*Test*	*Test statistic*	*p*	*Post-hoc comparisons*
**Number girls**	11	18	23	X^2^(2)	22.9	<.001	
**Age**	9.36 (.64)	9.67 (.59)	9.60 (.53)	F(2,89)	2.4	.096	
**Two CR RT task**							
*Omission errors*	8.14 (5.51)	4.21 (3.54)	3.32 (2.72)	F(2,89)	11.4	<.001	Sub<N, Sp
*Directional errors*	42.72 (17.0)	33.97 (16.37)	29.68(15.33)	F(2,89)	5.10	<.008	Sub<N, Sp
**Normal background noise condition**							
*Span*	2.64 (2.37)	3.97 (2.33)	5.32 (2.11)	F(2,89)	10.1	<.001	Sub< Ν <Sp
*Go/No-Go*	105.8 (33.1)	143.1 (39.3)	164.2 (27.9)	F(2,89)	22.9	<.001	Sub< Ν <Sp
*Verbal episodic mem.*	6.14 (2.63)	8.80 (2.59)	9.44 (2.90)	F(2,89)	13.1	<.001	Sub< Ν, Sp
*Verbal recognition*	25.8 (4.62)	28.7(3.64)	31.5 (4.41)	F(2,89)	13.2	<.001	Sub< Ν, Sp

*Note: Post-hoc comparisons are shown where p<.05; Sub = Sub-attentive, N = normal attention, Sp = Super-attentive.*

**Figure 1:**
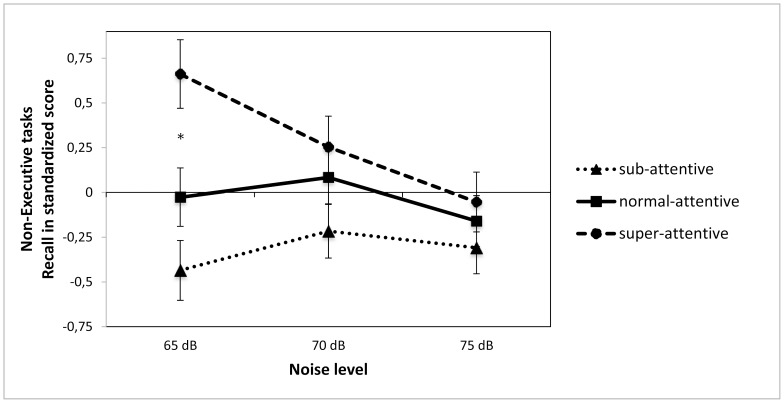
Performance on executive function tasks as a function of attention ability and noise level. Note: White noise levels were 65, 75, 85 dB; * indicates a significant difference between groups in the 65 dB condition (*F*(2,86) = 6.36, *p* = .003).

For non-EF there was a significant effect of group (*F*(2,86) = 5.69, *p*<.005), an effect of WN (*F*(2,86) = 3.09, *p* = .048) and a trend for a significant interaction between group and WN (*F*(4,172) = 2.19, *p* = .072), (see Figure 1). Adding moderate levels of WN had a different effect on the three groups. For the super-attentive there was a significant decline in performance (*F*(2,46) = 4.42, *p* = .018), for the average group there was a small, non-significant decline in performance (*F*(2,56) = 1.21, *p* = .305, *ns*), and for the sub-attentive group there was a small, non-significant increase in performance (*F*(2,66) = .385, *p* = .682, *ns*). This meant that the significant group difference seen at low WN levels (65dB *F*(2,86) = 8.18, *p* = .001) was no longer present at the moderate or the high WN levels (70dB *F*(2,86) = 1.88, *p* = .159, *ns*: 75dB *F*(2,86) = .587, *p* = .558, *ns*), (see Figure 1).

**Figure 2:**
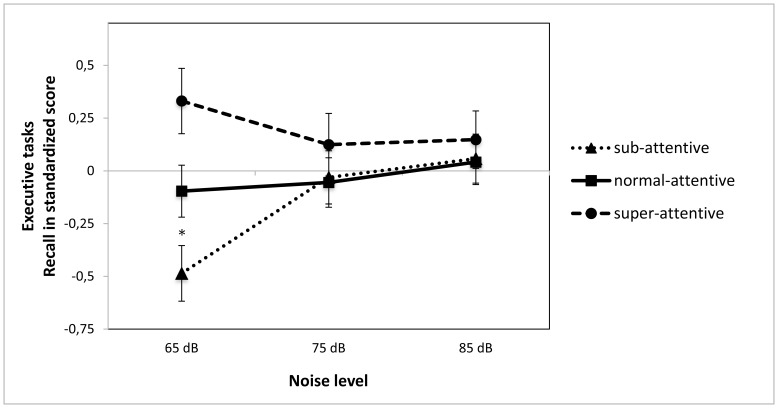
Performance on non-executive function tasks as a function of attention ability and noise level. Note: White noise levels were 65, 70, 75 dB, speech level ≈ 75 dB; * indicates a significant difference between groups in the 65 dB condition (*F*(2,86) = 8.18, *p* = .001).

For the EF tasks, although the pattern of performance changes was similar, the patterns of statistical significance were different (see Figure 2). There was only a trend for an effect of group (*F*(2,86) = 2.71, *p* = .073), and no significant effect of WN (*F*(2,86) = 1.89, *p* = 154, *ns*). There was however a significant interaction between group and WN (*F*(4,174) = 2.49, *p* = .045). Again, the shift from low to moderate levels of WN had a different effect on the three groups. The sub-attentive participants displayed a significant improvement in performance (*F*(2,66) = 7.39, *p* = .001), the average attention group showed a small, non-significant improvement in performance (*F*(2,56) = .230, *p* = .795, *ns*), and for the super-attentive group there was a small, non-significant decline in performance (*F*(2,46) = .202, *p* = .818, *ns*). Again, the significant group difference seen at low WN levels was no longer present at the moderate or the high WN levels (65dB *F*(2,86) = 6.36, *p* = .003∶75dB *F*(2,86) = .490, *p* = .615, *ns* : 85dB *F*(2,86) = .206, *p* = .814, *ns*), (see Figure 2).

**Figure 3:**
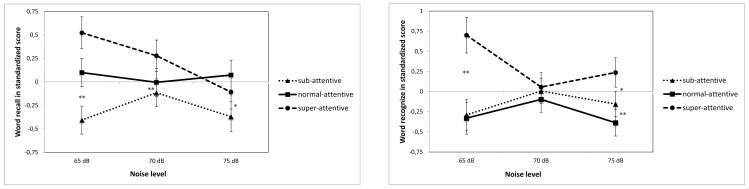
Figure 3A. Performance on Word recall task (non-executive) as a function of attention ability and noise level. Note: White noise levels were 65, 70, 75 dB, speech level ≈ 75 dB; * (p<.05) and ** (p<.001) indicates significant differences between super- and sub-attentive groups. **Figure 3B. Performance on Word recognize task (non-executive) as a function of attention ability and noise level.** Note: White noise levels were 65, 70, 75 dB, speech level ≈ 75 dB; * (p<.05) and ** (p<.001) indicates significant differences between super- and sub, normal-attentive groups in 65 and 75 dB conditions.

When looking at the tasks separately the non-executive, verbal task data, showed flowingly: In the Word recall task a two-way ANOVA indicated a trend towards an interaction between WM and group (F(4,172) = 2.12, *p = *.081) where the sub-attentive group improved their performance and the super-attentive got worse. There was a significant difference between groups (F(2,86) = 5.12, *p* = .007). A Bonferroni post hoc test showed a significant difference between the sub- and super-attentive groups in all three noise conditions, (see Figure 3A). In the Word recognition task the pattern was more marked, the difference between the sub- and normal-attentive groups and the super-attentive was significant in 65 dB (F(2,87) = 9.47, *p<.*001) but disappeared in the 70 dB condition (F(2,87) = 0.39, *p = *.679). A two-way ANOVA revealed a weak interaction between group and WN (F(4,172) = 2.22, *p* = .069); this interaction became however significant when the highest noise level (75 dB) was excluded (F(2,86) = 4.30, *p = *.017), (see Figure 3B).

**Figure 4:**
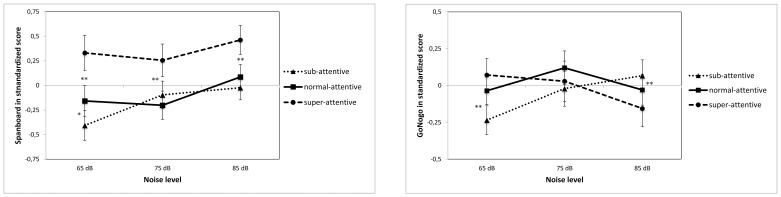
Figure 4A. Performance on Go/No-Go task (executive) as a function of attention ability and noise level. Note: White noise levels were 65, 75, 85 dB. ** (p<.001) indicates significant differences between super- and sub attentive in 65 and 85 dB conditions. **Figure 4B. Performance on Spanboard task (executive) as a function of attention ability and noise level.** Note: White noise levels were 65, 75, 85 dB. * (p<.05) and ** (p<.001) indicates significant differences between groups.

The results for the EF tasks displayed a somewhat different pattern. In the Spanboard task a two-way ANOVA gave away a positive main effect of noise (F(2,85) = 3.57, *p = *.032 and no interaction between WN and group. Performance levels differed over all noise conditions where the super-attentive group outperformed the other two (F(2,85) = 6.20, *p = *.003), (see Figure 4A). In the Go/No-Go task, however, the two-way ANOVA indicated a marginal interaction between WN and group (F(4,172) = 2.01, *p = *.095) but by excluding the normal-attentive group we got a perfect interaction between super- and sub-attentive groups (F(2,114) = 5.17, *p = *.007). In this task there was no difference in performance level between the groups (over noise conditions; F(2,86) = .224, *p = *.800), (see Figure 4B).

Notably, when looking at omission errors in the Go/No-Go task there was a large group difference, the sub-attentive group made far more omissions (M = 27,4) as compared to normal- (M = 14,4), and super-attentive groups (F(2,76) = 18.04, *p<*.001). Moreover, noise did exert a significant effect only on the sub-attentive group who improved considerably by noise exposure. A paired samples t-test gave away significant improvement both from 65 to 75 dB (t(31) = 2.16, *p = .*38) and from 65 to 85 dB (t(29) = 2,40, *p* = .023) for the sub attentive group. Noise exposure was however not sufficient to eliminate the differences in omission errors between the groups. No effect at all of noise was found on direction- or commission-errors in the Go/No-Go task for any group.

Table 2 shows the results of the Noise X Group X Task Type repeated measures ANOVA. A significant main effect of Group emerged, and again the super-attentive children performed better than the average attentive children, who outperformed the sub-attentive children (although the difference between the sub-attentive children and the normal attentive children did not reach statistical significance). There was also a significant interaction between WN and Group (Figure 1). The performance of the super-attentive group declined as the WN level increased to 75db (*t*(24) = 3.744, *p* = .001); the average attention group was unaffected (*t*(29) = .369 *p* = .715) while the sub-attentive group improved (*t*(34) = −2.247, *p* = .010).

**Table 2:** Main effects and interactions from a Group X Noise X Task Type repeated measures ANOVA.

	*df*	*F*	*p*
**Main Effects**			
Group	(1,85)	5.53	.006*
Noise	(1,85)	1.25	.267
Task Type	(1,85)	.521	.472
**Two Way Interactions**			
Noise × Group	(2, 85)	4.30	.017*
Task Type × Group	(2, 85)	.016	.984
Task Type × Noise	(2, 85)	4.94	.029*
**Three way interactions**			
Group × Noise × Task type	(2, 85)	.594	.554

*Note.* * = *p*<.05.

## Discussion

The current paper tested the hypothesis that increasing WN levels will differentially affect children’s performance as a function of their attention abilities (as rated by teachers). Based on the MBA model we predicted that high levels of WN will have a facilitative effect on task performance for sub-attentive children but will disrupt performance in super-attentive children. In line with this prediction, when all tasks were combined, adding WN disrupted the performance of the super-attentive group but improved the performance of the sub-attentive group. These findings extend those found in previous studies showing the differential effects of WN on sub- and normal attentive groups [7], [8] in a number of ways, including a super attentive group, extending the range of tasks, and extending the noise range.

First, by including a super-attentive group we were able to demonstrate that response to WN varied differentially according to the degree of attentiveness and to show that the children who were rated by their teachers as being the best able to pay attention were generally the most negatively affected by increasing levels of WN. In contrast to those who were least able to pay attention were those that gained the most benefit from increasing levels of WN. This suggests that the mechanisms governing the link between attentiveness, internal noise and external noise operate across the full range of attentional abilities.

Second, by extending the battery of tasks we were able to both: (i) examine the effects of WN on executive and non-executive tasks and (ii) improve the reliability of measurement of the impact of WN. Although there was an overall facilitative effect of moderate WN on sub-attentive children and a disruptive effect on the super-attentive children, the patterns of significance were somewhat different when the effects of EF and non-EF tasks were examined. For the non-EF tasks, the shift from low to moderate levels of WN was characterized by a significant decline in performance in the super-attentive group, whereas for the EF tasks this shift from low to moderate levels of WN was characterized by a significant improvement in performance in the sub-attentive group.

It is possible that the effect of WN on EF tasks is qualitatively different from the effect of WN on non-EF tasks. Thus, on the non-EF tasks moderate levels of WN are generally disruptive, particularly for the super attentive children, but sub-attentive individuals are protected from the disruptive effects of these levels of WN. In contrast, on the EF tasks moderate levels of WN actually have a facilitative effect on the sub-attentive children. However we cannot be certain of this as there are factors other than the EF/non EF distinction that differ between these two groups: the modality of the tasks (auditory delivered versus visually delivered) and the fact that the tasks were performed under different ranges of WN. It may be that the more fine grained but restricted range of WN levels adopted for the non-EF measures may allow the identification of different aspects of the noise-attention relationship, or that the auditory non-EF tasks were harder to perform under increasing WN levels, as the words became more difficult to hear. Of great importance to note is the cross modal nature of the WN effects seen for the EF tasks: auditory WN exerted an effect on the processing of visual stimuli, which has been shown earlier on signal detection [22] but here, to our knowledge for the first time, on EF as well. In the non-executive tasks noise and signal were exposed within the same modality and results could indicate that lower levels of WN are required for SR to occur within the same modality. Future research replicating these effects in non-auditory modalities, such as in a word recall tasks, in which the words are presented visually, will help to clarify this.

Third, we extended previous research by adding multiple noise levels that gave us more power to both: (i) examine the inverted U function of the effects of WN as predicted by SR models and (ii) identify more subtle differences in the WN-performance relationship and how this might change with increasing noise intensities. In this regard, the following findings were notable. Moving to the highest levels of WN intensity had little effect on the performance of any of the groups. There was little evidence for the inverted WN-performance U-function as predicted by the MBA model, where performance peaks at moderate noise levels but too much noise will cause performance to deteriorate. We further predicted that this inverted U shaped function of performance across different WN intensities would be right-shifted for sub-attentive children and left-shifted for super-attentive children compared to children with normal attention, as inattentive children should require more environmental WN than attentive children for optimal performance in cognitive tasks, and conversely these inattentive children should be able to tolerate greater levels of WN before performance deteriorates. However, the super attentive group showed a general pattern of decline across all three WN levels and the normal attentive group tended to exhibit consistent performance across the three noise levels, with only the sub-attentive group showing an improvement in performance as noise levels increased. In summary, the specific differential patterns of effects of increasing WN levels that we had predicted did not materialize; this could indicate that supra threshold SR acts differently compared to threshold SR [46].

It is not evident that the relation between the noise level and the outcome of high-level cognitive performance depicts an inverted U-curve as in signal detection tasks in threshold SR. When we are dealing with supra threshold SR the pattern might be biphasic instead; either there is an effect of noise or there is not. In cross modal SR, auditory noise on visual detection, using similar noise levels as in the present study, responses seems to mimic an inverted U-curve [22]. On the other hand when using stochastic vestibular stimulation to improve balance in healthy adults the effect was either present or not, even if stimulation thresholds differed between individuals [47].

When looking at the EF tasks separately we found that in the Go/No-Go task when inhibition is required there was an interaction between super- and sub attentive children. On the contrary, in the Spanboard task none of the groups was affected negatively by noise exposure and there was a just a positive main effect of noise. The Go/No-Go task put high demands on executive functioning, both the updating of information (inhibition) and maintenance of information, whereas the Spanboard task only put demands on maintenance. From this it can possibly be concluded that the higher the task demands are the more appropriate dopamine levels are required for a high performance [48]. Although noise is not found to increase dopamine levels per se, it looks like external noise in the nervous system acts in a similar fashion as dopamine release [49].

Regarding the two non-EF tasks, Word recall and Word recognition, there were tendencies towards an inverted U-function where performance peaked at 70 dB for the sub-attentive group whereas the super-attentive group got worse when exposed to increased noise levels. Lower levels of WN have to be used to find out if or where a peak would occur for the super-attentive group. The word recognition task represents long-term memory while it has an approximately 30 minutes delay between encoding and recall phases. It is therefore worth mentioning that words that were encoded in a moderately noisy environment were better recalled than the ones that were encoded at lower and higher noise levels for both the sub- and average-attentive groups. This may indicate that the positive effects of noise have not only an acute effect, but also a long-term one. This calls for further investigation.

An alternative explanation to consider is that rather than inducing SR, WN increased arousal in participants, which in turn affected information processing in different ways for the two groups. Such an explanation is consistent with the state regulation deficit model of ADHD [50] derived from cognitive energetic theory [51]. This theory posits that children with attention problems have difficulty modulating their levels of arousal and activation to adjust to changing circumstances and patterns of external stimulation – in particular they have difficulty maintaining arousal levels on challenging and boring tasks. It further predicts that these difficulties are alleviated by the addition of external stimulation. The finding in the current study that the performance of the sub-attentive group improved with moderate levels of WN is consistent with this prediction. Arousal might also offer an alternative explanation for the WN-related deterioration in the super-attentive children’s performance. It is possible that arousal levels are optimal for this group at the lowest noise level in the current study and that they become over-aroused by increases in WN. Alternatively it is possible that their performance deteriorates because they just find the noise annoying and distracting. This possibility needs to be investigated in future research using human and animal models, directly measuring known neural and physiological markers of arousal in experiments employing manipulations of alternative factors (event rate or stimulant medication) known to change energetic levels [49], [51]–[62].

Another possible explanation is that WN benefit results from auditory masking, as a masker different from the signal it can facilitate signal detection [63]. It has been shown that if the masker was predictable ADHD participants behave less impulsively [64] and exhibit improved signal detection [65]. Masking effects have been shown in both the visual [66] and tactile modalities [67]. In both SR and in masking, task irrelevant (meaningless) stimulation in different modalities increases the signal-to-noise ratio and thus improves performance on various tasks. To determine the importance of masking it would be useful to compare the effects of WM with sensory noise without sound masking properties like in vestibular noise [20], [68], [69].

Although our methodology improved on previous studies in a number of ways a number of limitations need to be acknowledged. *First*, we were unable to match the attention groups for sex of participant. Our super attentive group included far more girls (N = 23) than boys (N = 2) and the sub attentive group included more boys (N = 22) than girls (N = 14). We controlled for the effects of gender, using gender as a covariate, in all our analyses but it would be preferable to have the groups matched for gender to detect more clearly if the noise displays any gender differences. All patterns where the same for boys and girls, but we could discern a marginal effect of gender in the non-EF tasks. In the EF tasks using gender as a covariate did not change the noise x attention interaction at all but in the non-EF tasks the interaction got somewhat weaker when the effect of gender was used as a covariate. More statistic power is required to draw any conclusions from this and gender has to be addressed in a separate study for this purpose. Moreover, we screened a high number of children (N = 150) and it may be that preponderance of super-attentive individuals amongst girls is part of the normal variation within the classroom and this is highly interesting per se if it holds for true. *Second*, the experiments were conducted in a classroom setting where participants accomplished tests at their own school in groups of approximately five pupils, which could partly explain the poor results of the sub-attentive group – one might expect that they became more distracted by their class mates than the others. In order to investigate noise benefit in a lower range of WN we need to conduct lab studies where we can hold the ambient noise (NBN) at a constant very low level (<40 dB), as this will give us an opportunity to find out if normal- and super-attentive individuals could benefit from WN. *Third*, we failed to produce an inverted U-curve, as predicted, in any of the participating groups. We cannot rule out the possibility that adding more noise levels, in particular levels with lower intensity (at least 55 and 60 dB) might have produced improvements in the super-attentive group and thus produced an inverted U-curve in this group. SR research, that finds U-curves, is normally done in normal populations, the MBA-model [24] predicting a deviant pattern for inattentive (i.e. low dopamine) subjects. *Fourth*, the three WN levels were determined a-priori and were identical for all participants; it may be that if participants had been able to select their own levels of WN, they would be able to produce an optimal level and thus produce greater WN benefits. Future studies that allow participants to individually adjust the levels of WN exposure may help to clarify this effect further. *Fifth*, there were different patterns of WN effects for the different tasks, EF and non-EF. This may be a modality-related effect. There are few studies that investigate cross modal SR e.g. [22]. There are good reasons to believe that when you present target and noise in the same modality you need less noise to obtain the same SR-effect. For instance in our own data (manuscript in preparation) we find this effect with auditory noise on visual word recall tasks (instead of reading out words they are shown on a computer screen). Future studies should be designed to disentangle the effects of modality and task domains.

The beneficial effects of moderate levels of WN raise the question of its therapeutic potential for children with attention problems. Our data suggest that sub-attentive children benefit from the addition of moderate levels of auditory WN, particularly in tasks that require EFs. If this could be applied in a classroom setting, it may have important practical implications for improving the performance and outcome of children who typically find it difficult to pay attention. It is however unclear whether these effects will also apply to individuals with more extreme problems and clinical diagnoses. Previous research e.g. [4] has shown that background noise can have a beneficial effect on patients with ADHD. Given the fact that the sub-attentive children in the current study were selected because they were in the lowest quintile for attention ability it seems likely that these effects will also apply to clinical populations of patients with ADHD, and might offer an alternative therapy to children who do not respond to stimulant medication [70]. Upcoming research that employs clinical patients with ADHD and compares the efficacy of WN and stimulant medication will elucidate this. It will also be important to carefully tailor and monitor these interventions given the highly heterogeneous nature of ADHD. It is unlikely that all clinical cases will benefit from the same WN intensity, and some cases may not respond to noise at all.

In conclusion, we have demonstrated that adding moderate levels of WN could benefit the performance of sub-attentive children (as rated by their teachers), while similar changes can impair children with already good levels of attention. Increasing WN past moderate levels has little further effect.
